# Supplementary material for: End Sequence Analysis Toolkit (ESAT) expands the extractable information from single-cell RNA-seq data
Source: Genome Res. 2016 Oct;26(10):1397–410. doi: 10.1101/gr.207902.116 (PMC5052061; doi:10.1101/gr.207902.116)
Supplement: Supplemental Material [file supp_gr.207902.116_Supp_Materials.tar.gz › ESAT-esat_v0.1/doc/html/classbroad_1_1pda_1_1feature_1_1genome_1_1_chromosome.html]

ESAT: broad.pda.feature.genome.Chromosome Class Reference


ESAT


- Main Page
- Related Pages
- Packages
- Classes
- Files

- Class List
- Class Index
- Class Hierarchy
- Class Members

All Classes Namespaces Files Functions Variables Enumerator Pages

- broad
- pda
- feature
- genome
- Chromosome

Classes |
Public Member Functions |
Static Public Member Functions |
List of all members

broad.pda.feature.genome.Chromosome Class Reference

Collaboration diagram for broad.pda.feature.genome.Chromosome:

[legend]

|  |  |
| --- | --- |
| Classes | |
| class | **AgpEntry** |
|  | |
| class | **AgpEntryFactory** |
|  | |

|  |  |
| --- | --- |
| Public Member Functions | |
|  | Chromosome (final String name) |
|  | |
|  | Chromosome (String name, String agpFile) throws Exception |
|  | |
| boolean | isSexChromosome () |
|  | |
| Annotation | getShortArm () |
|  | |
| boolean | isAutosome () |
|  | |
| String | getName () |
|  | |
| String | getSymbol () |
|  | |
| String | getChromosomeNumber () |
|  | |
| int | compareTo (Object o) |
|  | |
| long | getUngappedSize () |
|  | |
| void | setSequenceFile (File file) |
|  | |
| int | length () |
|  | |
| List< AgpEntry > | gaps () |
|  | |
| SequenceRegion | drawRandomRegion (int size) |
|  | |
| List<?extends GenomicAnnotation > | shuffle (GenomicAnnotation toShuffle, List<?extends GenomicAnnotation > toAvoid) |
|  | |
| List<?extends Annotation > | shuffleWithinValid (Annotation toShuffle, List<?extends Annotation > valid) |
|  | |
| List<?extends GenomicAnnotation > | shuffle (List<?extends GenomicAnnotation > toShuffle, List<?extends GenomicAnnotation > toAvoid) |
|  | |
| List<?extends GenomicAnnotation > | shuffleWithinValid (List<?extends Annotation > toShuffle, List<?extends Annotation > valid) |
|  | |
| void | getRegion (SequenceRegion region) |
|  | |
| void | getRegion (SequenceRegion region, boolean softmask) |
|  | |
| void | getRegion (SequenceRegion region, boolean softmask, Map< String, IntervalTree< Alignments >> okRepeats) |
|  | |
| List< SequenceRegion > | getRegions (List<?extends GenomicAnnotation > annotations, int padding) throws IOException |
|  | |
| void | extractRegions (List<?extends SequenceRegion > regions) throws IOException |
|  | |
| void | extractRegion (SequenceRegion region) throws IOException |
|  | |
| void | loadSequence () throws IOException |
|  | |
| void | unloadSequence () |
|  | |
| Sequence | getSequence () |
|  | |
| void | insert (SequenceRegion region, LightweightGenomicAnnotation insertPoint) |
|  | |
| void | delete (SequenceRegion region) |
|  | |
| void | invert (SequenceRegion region) |
|  | |

|  |  |
| --- | --- |
| Static Public Member Functions | |
| static boolean | isSexChromosome (String name) |
|  | |
| static boolean | isAutosome (String name) |
|  | |
| static String | getChromosomeNumber (String name) |
|  | |
| static int | compareNames (String name1, String name2) |
|  | |

## Detailed Description

Represents a chromosome in a genome

Author
:   engreitz

## Constructor & Destructor Documentation

|  |  |  |  |  |  |
| --- | --- | --- | --- | --- | --- |
| broad.pda.feature.genome.Chromosome.Chromosome | ( | final String | *name* | ) |  |

Parameters
:   |  |  |
    | --- | --- |
    | The | name of the chromosome e.g. "chr12" |

Here is the call graph for this function:

Here is the caller graph for this function:

|  |  |  |  |
| --- | --- | --- | --- |
| broad.pda.feature.genome.Chromosome.Chromosome | ( | String | *name*, |
|  |  | String | *agpFile* |
|  | ) |  | throws Exception |

Parameters
:   |  |  |
    | --- | --- |
    | The | name of the chromosome e.g. "chr12" |
    | An | AGP file describing the chromosome assembly |

Here is the call graph for this function:

## Member Function Documentation

|  |  |  |  |
| --- | --- | --- | --- |
| static int broad.pda.feature.genome.Chromosome.compareNames | ( | String | *name1*, |
|  |  | String | *name2* |
|  | ) |  |  |

static

Here is the call graph for this function:

|  |  |  |  |  |  |
| --- | --- | --- | --- | --- | --- |
| int broad.pda.feature.genome.Chromosome.compareTo | ( | Object | *o* | ) |  |

Here is the call graph for this function:

|  |  |  |  |  |  |
| --- | --- | --- | --- | --- | --- |
| void broad.pda.feature.genome.Chromosome.delete | ( | SequenceRegion | *region* | ) |  |

Here is the call graph for this function:

|  |  |  |  |  |  |
| --- | --- | --- | --- | --- | --- |
| SequenceRegion broad.pda.feature.genome.Chromosome.drawRandomRegion | ( | int | *size* | ) |  |

Here is the call graph for this function:

|  |  |  |  |  |  |
| --- | --- | --- | --- | --- | --- |
| void broad.pda.feature.genome.Chromosome.extractRegion | ( | SequenceRegion | *region* | ) | throws IOException |

Here is the call graph for this function:

|  |  |  |  |  |  |
| --- | --- | --- | --- | --- | --- |
| void broad.pda.feature.genome.Chromosome.extractRegions | ( | List<?extends SequenceRegion > | *regions* | ) | throws IOException |

Here is the call graph for this function:

Here is the caller graph for this function:

|  |  |  |  |  |
| --- | --- | --- | --- | --- |
| List<AgpEntry> broad.pda.feature.genome.Chromosome.gaps | ( |  | ) |  |

Here is the call graph for this function:

Here is the caller graph for this function:

|  |  |  |  |  |
| --- | --- | --- | --- | --- |
| String broad.pda.feature.genome.Chromosome.getChromosomeNumber | ( |  | ) |  |

Here is the caller graph for this function:

|  |  |  |  |  |  |
| --- | --- | --- | --- | --- | --- |
| static String broad.pda.feature.genome.Chromosome.getChromosomeNumber | ( | String | *name* | ) |  |

static

|  |  |  |  |  |
| --- | --- | --- | --- | --- |
| String broad.pda.feature.genome.Chromosome.getName | ( |  | ) |  |

Here is the caller graph for this function:

|  |  |  |  |  |  |
| --- | --- | --- | --- | --- | --- |
| void broad.pda.feature.genome.Chromosome.getRegion | ( | SequenceRegion | *region* | ) |  |

Here is the caller graph for this function:

|  |  |  |  |
| --- | --- | --- | --- |
| void broad.pda.feature.genome.Chromosome.getRegion | ( | SequenceRegion | *region*, |
|  |  | boolean | *softmask* |
|  | ) |  |  |

|  |  |  |  |
| --- | --- | --- | --- |
| void broad.pda.feature.genome.Chromosome.getRegion | ( | SequenceRegion | *region*, |
|  |  | boolean | *softmask*, |
|  |  | Map< String, IntervalTree< Alignments >> | *okRepeats* |
|  | ) |  |  |

|  |  |  |  |
| --- | --- | --- | --- |
| List<SequenceRegion> broad.pda.feature.genome.Chromosome.getRegions | ( | List<?extends GenomicAnnotation > | *annotations*, |
|  |  | int | *padding* |
|  | ) |  | throws IOException |

Here is the call graph for this function:

|  |  |  |  |  |
| --- | --- | --- | --- | --- |
| Sequence broad.pda.feature.genome.Chromosome.getSequence | ( |  | ) |  |

|  |  |  |  |  |
| --- | --- | --- | --- | --- |
| Annotation broad.pda.feature.genome.Chromosome.getShortArm | ( |  | ) |  |

|  |  |  |  |  |
| --- | --- | --- | --- | --- |
| String broad.pda.feature.genome.Chromosome.getSymbol | ( |  | ) |  |

Here is the call graph for this function:

Here is the caller graph for this function:

|  |  |  |  |  |
| --- | --- | --- | --- | --- |
| long broad.pda.feature.genome.Chromosome.getUngappedSize | ( |  | ) |  |

Here is the call graph for this function:

|  |  |  |  |
| --- | --- | --- | --- |
| void broad.pda.feature.genome.Chromosome.insert | ( | SequenceRegion | *region*, |
|  |  | LightweightGenomicAnnotation | *insertPoint* |
|  | ) |  |  |

Here is the call graph for this function:

|  |  |  |  |  |  |
| --- | --- | --- | --- | --- | --- |
| void broad.pda.feature.genome.Chromosome.invert | ( | SequenceRegion | *region* | ) |  |

Here is the call graph for this function:

|  |  |  |  |  |
| --- | --- | --- | --- | --- |
| boolean broad.pda.feature.genome.Chromosome.isAutosome | ( |  | ) |  |

Here is the caller graph for this function:

|  |  |  |  |  |  |
| --- | --- | --- | --- | --- | --- |
| static boolean broad.pda.feature.genome.Chromosome.isAutosome | ( | String | *name* | ) |  |

static

Here is the call graph for this function:

|  |  |  |  |  |
| --- | --- | --- | --- | --- |
| boolean broad.pda.feature.genome.Chromosome.isSexChromosome | ( |  | ) |  |

|  |  |  |  |  |  |
| --- | --- | --- | --- | --- | --- |
| static boolean broad.pda.feature.genome.Chromosome.isSexChromosome | ( | String | *name* | ) |  |

static

|  |  |  |  |  |
| --- | --- | --- | --- | --- |
| int broad.pda.feature.genome.Chromosome.length | ( |  | ) |  |

Here is the call graph for this function:

Here is the caller graph for this function:

|  |  |  |  |  |
| --- | --- | --- | --- | --- |
| void broad.pda.feature.genome.Chromosome.loadSequence | ( |  | ) | throws IOException |

Here is the call graph for this function:

Here is the caller graph for this function:

|  |  |  |  |  |  |
| --- | --- | --- | --- | --- | --- |
| void broad.pda.feature.genome.Chromosome.setSequenceFile | ( | File | *file* | ) |  |

Here is the caller graph for this function:

|  |  |  |  |
| --- | --- | --- | --- |
| List<? extends GenomicAnnotation> broad.pda.feature.genome.Chromosome.shuffle | ( | GenomicAnnotation | *toShuffle*, |
|  |  | List<?extends GenomicAnnotation > | *toAvoid* |
|  | ) |  |  |

|  |  |  |  |
| --- | --- | --- | --- |
| List<? extends GenomicAnnotation> broad.pda.feature.genome.Chromosome.shuffle | ( | List<?extends GenomicAnnotation > | *toShuffle*, |
|  |  | List<?extends GenomicAnnotation > | *toAvoid* |
|  | ) |  |  |

Randomizes (using a quasi uniform distribution) a list of genomic annotations

Parameters
:   |  |  |
    | --- | --- |
    | toShuffle | List of genomic annotations to shuffle |
    | toAvoid | regions to avoid placing a shuffled annotation. |

Returns

Here is the call graph for this function:

|  |  |  |  |
| --- | --- | --- | --- |
| List<? extends Annotation> broad.pda.feature.genome.Chromosome.shuffleWithinValid | ( | Annotation | *toShuffle*, |
|  |  | List<?extends Annotation > | *valid* |
|  | ) |  |  |

Here is the caller graph for this function:

|  |  |  |  |
| --- | --- | --- | --- |
| List<? extends GenomicAnnotation> broad.pda.feature.genome.Chromosome.shuffleWithinValid | ( | List<?extends Annotation > | *toShuffle*, |
|  |  | List<?extends Annotation > | *valid* |
|  | ) |  |  |

Here is the call graph for this function:

|  |  |  |  |  |
| --- | --- | --- | --- | --- |
| void broad.pda.feature.genome.Chromosome.unloadSequence | ( |  | ) |  |

---

The documentation for this class was generated from the following file:

- java/broad/pda/feature/genome/Chromosome.java


---

Generated on Fri Aug 15 2014 15:10:59 for ESAT by  

 1.8.7
